# Supplementary material for: Multifunctional antiferromagnetic materials with giant piezomagnetism and noncollinear spin current
Source: Nat Commun. 2021 May 14;12:2846. doi: 10.1038/s41467-021-23127-7 (PMC8121910; doi:10.1038/s41467-021-23127-7)
Supplement: Supplementary file 1 — Supplementary Information [file 41467_2021_23127_MOESM1_ESM.pdf]

Supplementary Information for
Multifunctional Antiferromagnetic Materials with Giant Piezomagnetism and Noncollinear Spin Current

Hai-Yang Ma[1], Mengli Hu[2], Nana Li[2], Jian-Peng Liu[2], Wang Yao[3], Jin-Feng Jia[1,4], and Junwei Liu[2]

Three sections are contained in this Supplementary Information (SI), Section I is on the details of the general theory of SVL-induced piezomagnetic effect and DC spin current. Section II and III are the results of the first-principles calculations. Section II is on the bulk properties of V_2Se_2O which includes Fig. S1-9 and Table S1. Section III is about the properties of the monolayer, which includes Fig. S10-23 and Table S2-3.

Fig. S1 Optimized primitive cell of bulk V_2Se_2O.
Fig. S2 Different magnetic order of bulk V_2Se_2O in a conventional cell.
Fig. S3 Total energy difference between different magnetic orders of the bulk.
Fig. S4 Same as Fig. 3 but with different method.
Fig. S5 Phonon spectrum of bulk V_2Se_2O.
Table S1 Optimized lattice constants of bulk V_2Se_2O using different functionals.
Fig. S6 Band structures of bulk V_2Se_2O at different U.
Fig. S7 Band structures of bulk V_2Se_2O with different magnetic orders.
Fig. S8 Band structures of V_2X_2O (X = O, S, Se or Te).
Fig. S9 Band structures of bulk V_2Te_2O with different magnetic orders.
Fig. S10 Total energy difference between different magnetic orders of the monolayer.
Fig. S11 Local magnetic moments of the V atoms in the monolayer.
Table S2 Optimized lattice constants of monolayer V_2Se_2O using different functionals.
Fig. S12 Magnetic properties of monolayer V_2Se_2O.
Fig. S13 Site-projected DOS of monolayer V_2Se_2O.
Fig. S14 Schematic of different magnetic orders of monolayer V_2Se_2O.
Table S3 Calculated Heisenberg exchange parameters of monolayer V_2Se_2O.
Fig. S15 Band structures of monolayer V_2Se_2O at different U.
Fig. S16 Total DOS and HSE06 band structures of of monolayer V_2Se_2O.
Fig. S17-18 Projected band structures of monolayer V_2Se_2O.
Fig. S19 Band structures of monolayer V_2Se_2O with and without SOC.
Fig. S20 Band structures at different uniaxial strain of monolayer V_2Se_2O.
Fig. S21 Effective masses of the valence valleys extrema as a function of uniaxial strain.
Fig. S22 Magnetization induced by uniaxial strain along **y** direction for monolayer V_2Se_2O.
Fig. S23 Dynamic stability and mechanical properties of monolayer V_2Se_2O.

CONTENTS

I. General theory of C-paired SVL and its unique properties 2
  A. General theory of C-paired SVL 2
  B. Strain induced valley polarization and piezomagnetic effect 2
  C. DC spin current 4

II. Properties of bulk V_2Se_2O 6
  A. Ground state determination 6
  B. Lattice constants 6
  C. Phonon spectrum and band structures 7
  D. Band structures of different magnetic orders 7
  E. Band structures of V_2X_2O (X = O, S, Se or Te) 8
  F. Band structures of bulk V_2Te_2O with different magnetic orders 8

III. Properties of monolayer V_2Se_2O 8
  A. Ground state and magnetic order 8
  B. Lattice constants 9
  C. Magnetic properties 9
    1. Crystal-field-theory analysis 9
    2. Site-projected DOS in local coordinates 10
    3. Superexchange interactions 11
    4. Parameters of the Heisenberg model 11
    5. Discussion 12
  D. Band structures of monolayer V_2Se_2O obtained with different functionals 12
  E. Projected band structures 13
  F. Strain effects on the band structures and effective mass 13
  G. Magnetization induced by uniaxial strain along **b** direction 14
  H. Calculations of the DC spin current of monolayer V_2Se_2O 15
  I. Dynamical stability and mechanical

properties 15
References 16

I. GENERAL THEORY OF C-PAIRED SVL AND ITS UNIQUE PROPERTIES

A. General theory of C-paired SVL

Without loss of generality, consider a 2D anti-ferromagnetic (AFM) system with two sublattices A and B with the local magnetic moments $\mathbf{M}_{R_A,R_B}$. By definition of AFM, the total magnetic moment should be zero, i.e. $\mathbf{M}_{R_A} + \mathbf{M}_{R_B} = 0$. It means there must be a symmetry operation C connecting two sublattices as $\mathbf{R}_A = C\mathbf{R}_B$, $\vec{\sigma}_{R_A} = C^\dagger \vec{\sigma}_{R_B} C$ and $\mathbf{M}_{R_A} = C^\dagger \mathbf{M}_{R_B} C$, where $\mathbf{R}_{A,B}$ are the coordinates and $\vec{\sigma}_{R_A,R_B}$ are local spin operator. By definition, the local moments can be calculated as $\mathbf{M}_{R_A,R_B} = \sum_n \int_\Omega \langle \phi_n(\mathbf{k}) | \vec{\sigma}_{R_A,R_B} | \phi_n(\mathbf{k}) \rangle \, d\mathbf{k}$, where $\sum_n \int_\Omega$ means the integral for all the occupied states. On the other hand, due to the symmetry operation,

$$\begin{aligned}
\mathbf{M}_{R_A} &= C^\dagger \mathbf{M}_{R_B} C \\
&= \sum_n \int_\Omega C^\dagger \langle \phi_n(\mathbf{k}) | \vec{\sigma}_{R_B} | \phi_n(\mathbf{k}) \rangle \, C d\mathbf{k} \\
&= \sum_n \int_\Omega \left\langle \phi_n(C\mathbf{k}) | C^\dagger \vec{\sigma}_{R_B} C | \phi_n(C\mathbf{k}) \right\rangle d\mathbf{k} \\
&= \sum_n \int_\Omega \langle \phi_n(C\mathbf{k}) | \vec{\sigma}_{R_A} | \phi_n(C\mathbf{k}) \rangle \, d\mathbf{k}.
\end{aligned} \tag{1}$$

To satisfy $\mathbf{M}_{R_A} = -\mathbf{M}_{R_B}$ in general, one can get

$$\langle \phi_n(C\mathbf{k}) | \vec{\sigma}_{R_A} | \phi_n(C\mathbf{k}) \rangle = - \langle \phi_n(\mathbf{k}) | \vec{\sigma}_{R_B} | \phi_n(\mathbf{k}) \rangle \, . \tag{2}$$

And similarly, one can get

$$\langle \phi_n(C\mathbf{k}) | \vec{\sigma}_{R_B} | \phi_n(C\mathbf{k}) \rangle = - \langle \phi_n(\mathbf{k}) | \vec{\sigma}_{R_A} | \phi_n(\mathbf{k}) \rangle \, . \tag{3}$$

Then, we can get

$$\begin{aligned}
\mathbf{M}_{C\mathbf{k}} &= \sum_{R_A} \langle \phi_n(C\mathbf{k}) | \sigma_{R_A} | \phi_n(C\mathbf{k}) \rangle + \sum_{R_B} \langle \phi_n(C\mathbf{k}) | \sigma_{R_B} | \phi_n(C\mathbf{k}) \rangle \\
&= -\sum_{R_A} \langle \phi_n(\mathbf{k}) | \sigma_{R_A} | \phi_n(\mathbf{k}) \rangle - \sum_{R_B} \langle \phi_n(\mathbf{k}) | \sigma_{R_B} | \phi_n(\mathbf{k}) \rangle \\
&= -\mathbf{M}_{\mathbf{k}}
\end{aligned} \tag{4}$$

It means that the spin-polarization for states at $\mathbf{k}$ and $C\mathbf{k}$ are always opposite. If there are two valleys $\mathbf{K}$ and $C\mathbf{K}$ at different positions of the momentum space, the symmetry C will enable a strict spin-valley locking. This can be easily generated to the case of AFM with multiple sublattices.

B. Strain induced valley polarization and piezomagnetic effect

The $\mathbf{k} \cdot \mathbf{p}$ equation of a crystal is read as[1–3]

$$H u^0_{n\mathbf{k}}(\mathbf{x}) = E^0_n(\mathbf{k}) u^0_{n\mathbf{k}}(\mathbf{x}), \tag{5}$$

where

$$H = H_0 + H_{\mathbf{k}} + H_{\mathbf{k}\cdot\mathbf{p}}, \tag{6}$$

here we ignore the relativistic effects and

$$\begin{aligned}
H_0 &= \frac{p^2}{2m} \\
H_{\mathbf{k}} &= \frac{\hbar k^2}{2m} + V_0(\mathbf{x}) \\
H_{\mathbf{k}\cdot\mathbf{p}} &= \frac{\hbar}{m} \mathbf{k} \cdot \mathbf{p}
\end{aligned} \tag{7}$$

for the cell periodic part of the Bloch wave function. Under homogeneous strain, we adopt the methods proposed

by Pikus and Bir[1] by which we can perform perturbation on the unstrained wave function $u_{n\mathbf{k}}(\mathbf{x})$. With this method we have

$$\begin{aligned} \mathbf{x}' &= (1-\epsilon)\mathbf{x} \\ \mathbf{p}' &= (1+\epsilon)\mathbf{p} \\ V(\mathbf{x}',\epsilon) &= V_0(\mathbf{x}) + \sum_{ij} V_{ij}(\mathbf{x})\epsilon_{ij} \ , \end{aligned} \tag{8}$$

where $V_{ij}(\mathbf{x}) = \partial V_0(\mathbf{x})/\partial\epsilon_{ij}$. The additional terms to the Hamiltonian induced by the strain up to its first order is

$$D = D_0 + D_{\mathbf{k}\cdot\mathbf{p}}, \tag{9}$$

with

$$\begin{aligned} D_0 &= \sum_{ij}\Big[-\frac{p_i p_j}{m} + V_{ij}(\mathbf{x})\Big]\epsilon_{ij} \\ D_{\mathbf{k}\cdot\mathbf{p}} &= -\frac{\hbar}{m}\sum_{ij} k_i\epsilon_{ij}p_j \ . \end{aligned} \tag{10}$$

Here the superscripts $'$ are omitted. The energy gain at the valley extrema (assume to be non-degenerate) then is

$$\Delta E = \langle u_n|D_0 + D_{\mathbf{k}\cdot\mathbf{p}}|u_n\rangle \ , \tag{11}$$

where u_n refers to wave function of nth band. This form is usually refers to the deformation potential theory[1] if rewrite as

$$\Delta E = \sum_{ij} D_{ij}\epsilon_{ij} \ . \tag{12}$$

where D is the tensor of deformation potential, ϵ is the strain tensor. For 2D SVL material having two valleys in the first BZ which are related by a mirror operation

$$M_\phi = \begin{pmatrix} -\cos(2\phi) & -\sin(2\phi) \\ -\sin(2\phi) & \cos(2\phi) \end{pmatrix} \ ,$$

where ϕ is the angle between the mirror plane and $\mathbf{x}$ axis. Choosing the coordinate so that major axis of valley K coincide with the $\mathbf{x}$ axis. Upon external strain, the energy shift of valley K is

$$\Delta E_K = D_{xx}\epsilon_{xx} + D_{yy}\epsilon_{yy} + 2D_{xy}\epsilon_{xy} \ . \tag{13}$$

For valley K', using the coordinate transformation $D_{K'} = M_\phi D_K M_\phi^{-1}$, explicitly

$$\begin{aligned} D^{11}_{K'} &= D_{xx}\cos^2(2\phi) + D_{yy}\sin^2(2\phi) + D_{xy}\sin(4\phi) \\ D^{12}_{K'} &= (D_{xx}-D_{yy})\sin(4\phi)/2 - D_{xy}\cos(4\phi) \\ D^{21}_{K'} &= (D_{xx}-D_{yy})\sin(4\phi)/2 - D_{xy}\cos(4\phi) \\ D^{22}_{K'} &= D_{xx}\sin^2(2\phi) + D_{yy}\cos^2(2\phi) - D_{xy}\sin(4\phi) \ , \end{aligned} \tag{14}$$

the energy shift of valley K' can be calculated as

$$\begin{aligned} \Delta E_{K'} = {} & [D_{xx}\cos^2(2\phi) + D_{yy}\sin^2(2\phi) + D_{xy}\sin(4\phi)]\epsilon_{xx} \\ & + 2[(D_{xx}-D_{yy})\sin(4\phi)/2 - D_{xy}\cos(4\phi)]\epsilon_{xy} \\ & + [D_{xx}\sin^2(2\phi) + D_{yy}\cos^2(2\phi) - D_{xy}\sin(4\phi)]\epsilon_{yy} \ . \end{aligned} \tag{15}$$

The relative energy shift of the two valley then is

$$\begin{aligned} \delta E = {} & \Delta E_K - \Delta E_{K'} \\ = {} & [(D_{xx}-D_{yy})\sin^2(2\phi) - D_{xy}\sin(4\phi)]\epsilon_{xx} + \\ & [(D_{yy}-D_{xx})\sin^2(2\phi) + D_{xy}\sin(4\phi)]\epsilon_{yy} + \\ & [(D_{yy}-D_{xx})\sin(4\phi) + 4D_{xy}\cos^2(2\phi)]\epsilon_{xy} \ . \end{aligned} \tag{16}$$

Now considering uniaxial strain applied along the direction having the angle θ with respect to the $\mathbf{x}$ axis, the matrix of the strain tensor can be obtained as

$$\epsilon = R(\theta)\begin{pmatrix} \epsilon_\theta & 0 \\ 0 & 0 \end{pmatrix} R^{-1}(\theta) = \begin{pmatrix} \cos^2(\theta) & \sin(2\theta)/2 \\ \sin(2\theta)/2 & \sin^2(\theta) \end{pmatrix}\epsilon_\theta \ ,$$

here we denote ϵ_{xx} with ϵ_θ, $R(\theta)$ is the 2D rotation matrix. Substituting it to Eq. (12), the relative energy shift of the two valleys is

$$\begin{aligned} \delta E(\theta) = {} & \Delta E_K(\theta) - \Delta E_{K'}(\theta) \\ = {} & [(D_{xx}-D_{yy})\sin(2\phi) - 2D_{xy}\cos(2\phi)] \\ & \sin(2\phi - 2\theta)\epsilon_\theta \\ = {} & \gamma\sin(2\phi - 2\theta)\epsilon_\theta \end{aligned} \tag{17}$$

with

$$\gamma = (D_{xx}-D_{yy})\sin(2\phi) - 2D_{xy}\cos(2\phi) \ . \tag{18}$$

Now we check the results with

$$\Delta E_K(\theta) - \Delta E_{K'}(\theta) = \Delta E_{K'}(M_\phi\theta) - \Delta E_K(M_\phi\theta), \tag{19}$$

as required by the mirror symmetry, this is equivalent to show that $\delta E(\theta) = -\delta E(M_\phi\theta) = -\delta E(2\phi - \theta)$ as

$$\begin{aligned} \delta E(2\phi-\theta) = {} & \{(D_{xx}-D_{yy})\sin(2\phi)\sin[2\phi - 2(2\phi-\theta)] - \\ & 2D_{xy}\cos(2\phi)\sin[2\phi - 2(2\phi-\theta)]\}\epsilon_\theta \\ = {} & -[(D_{xx}-D_{yy})\sin(2\phi)\sin(2\phi-2\theta) - \\ & 2D_{xy}\cos(2\phi)\sin(2\phi-2\theta)]\epsilon_\theta \\ = {} & -\delta E(\theta) \ , \end{aligned} \tag{20}$$

which verified the results.

From the above discussions we know that unaxial stain may cause nonzero relative energy shift between the two valleys. Therefore, if further dopes the sample, uncompensated electrons or holes with specific spin should appear as

$$M = \int_{-\infty}^{E_f(n)} [\rho^\uparrow(\epsilon) - \rho^\downarrow(\epsilon)]dE \ , \tag{21}$$

where E_f is the fermi level after doping, n is the doping density, $\rho^{\uparrow(\downarrow)}$ is the spin-up (down) part of the density of states (DOS), which is dependent on the external

strain ϵ. Around the valley extrema, it may be taken as a constant ρ for 2D parabolic band and the magnetic moments per electron are taken as 1. After carefully analyzing the behaviors of the induced magnetizations under different doping concentration and uniaxial strain, the explicit form of M under specific strain while varies with the doping concentration n is

$$M(n,\theta,\epsilon_\theta)=\begin{cases}-np, & 0\le n<|m|\epsilon_\theta\\ -m\epsilon_\theta, & n\ge |m|\epsilon_\theta\end{cases}, \tag{22}$$

where $m=\rho\gamma\sin(2\phi-2\theta)$, $p=sign[\gamma\sin(2\phi-2\theta)]$. And varies with strain ϵ_θ while at finite doping concentration

$$M(n,\theta,\epsilon_\theta)=\begin{cases}-m\epsilon_\theta, & 0\le \epsilon_\theta<n/|m|\\ -np, & \epsilon_\theta\ge n/|m|\end{cases}, \tag{23}$$

the piezomagnetic effect is obvious.

Now we apply the above discussions to the valence valleys of monolayer V_2Se_2O, whose mirror plane is long the diagonal $\phi=\pm\pi/4$ and $\pm 3\pi/4$. Upon uniaxial strain along $\mathbf{x}$ direction, the energy shifts of the X or Y valley are

$$\begin{aligned}\Delta E_X &= D_{xx}\epsilon_{xx}\\ \Delta E_Y &= D_{yy}\epsilon_{xx}\ .\end{aligned} \tag{24}$$

Therefore, the relative energy shift between the two valleys is

$$\delta E=\Delta E_X-\Delta E_Y=(D_{xx}-D_{yy})\epsilon_{xx}\ . \tag{25}$$

This is nothing but the basic law of the PZM in hole-doped monolayer V_2Se_2O if further proceeding to the DOS difference.

C. DC spin current

From Bolzman equation the DC conductivity is read as[4]:

$$\sigma=\sum_n\Big[e^2\int\frac{d^3k}{4\pi^3}\tau_n(E_n(\mathbf{k}))\mathbf{v}_n(\mathbf{k})\mathbf{v}_n(\mathbf{k})\Big(-\frac{\partial f}{\partial E}\Big)_{E=E_n(\mathbf{k})}\Big], \tag{26}$$

where n is the band index; $E_n(\mathbf{k})$ is the band energy; $\tau_n(E_n(\mathbf{k}))$ is the relaxation time, which can be taken as a constant; $\mathbf{v}_n(\mathbf{k})=\frac{\partial E_n(\mathbf{k})}{\hbar\partial\mathbf{k}}$ is the velocity and $f(E)=1/(e^{[E-E_f]/k_BT}+1)$ is the Fermi-Dirac distribution function. Explicitly

$$\begin{aligned}\sigma&=\sum_n\Big[-e^2\tau\int\frac{d^3k}{4\pi^3}\frac{\partial E_n(\mathbf{k})}{\hbar\partial\mathbf{k}}\frac{\partial E_n(\mathbf{k})}{\hbar\partial\mathbf{k}}\frac{\partial f(E_n(\mathbf{k}))}{\partial E_n(\mathbf{k})}\Big]\\ &=\sum_n\Big[-e^2\tau\int\frac{d^3k}{4\pi^3}\frac{1}{\hbar^2}\frac{\partial E_n(\mathbf{k})}{\partial\mathbf{k}}\frac{\partial f(E_n(\mathbf{k}))}{\partial\mathbf{k}}\Big],\end{aligned} \tag{27}$$

Integrating by part and using the fact that the boundary integral must vanish[5], we obtain

$$\sigma=\sum_n\Big[e^2\tau\int\frac{d^3k}{4\pi^3}f(E_n(\mathbf{k}))\frac{1}{\hbar^2}\frac{\partial^2E_n(\mathbf{k})}{\partial\mathbf{k}^2}\Big]\ . \tag{28}$$

Written the index explicitly

$$\sigma_{ij}=\sum_n\Big[e^2\tau\int\frac{d^3k}{4\pi^3}f(E_n(\mathbf{k}))\frac{1}{\hbar^2}\frac{\partial^2E_n(\mathbf{k})}{\partial k_i\partial k_j}\Big], \tag{29}$$

where $i,j=x,y$ or z. For 2D material, by changing the metric of the integral $\int\frac{d^3k}{8\pi^3}\to\int\frac{d^2k}{4\pi^2}$, we obtain the conductivity tensor

$$\sigma_{ij}=\sum_n\Big[e^2\tau\int\frac{d^2k}{4\pi^2}f(E_n(\mathbf{k}))\frac{1}{\hbar^2}\frac{\partial^2E_n(\mathbf{k})}{\partial k_i\partial k_j}\Big], \tag{30}$$

where $i,j=x$ or y. The additional 1/2 is from spin since the valleys are spin-polarized.

In low-energy limit, the energy of the band extrema (assumes to be non-degenerate) can be expanded in quadratic terms of $\mathbf{k}$. For materials with two valleys in the first BZ which are related by mirror operation M_ϕ, here ϕ is the angle between the mirror plane and $\mathbf{x}$ axis. One can always choose the coordinate to make the major axis of one valley coincides with the $\mathbf{x}$ axis as

$$E_K=\frac{\hbar^2}{2m_1}k_x^2+\frac{\hbar^2}{2m_2}k_y^2\ . \tag{31}$$

Of the another valley lies at $K'=C^{-1}K$, where $C=M_\phi$, the energy dispersions of vallye K' are

$$\begin{aligned}E_{K'}=&\frac{\hbar^2}{2m_1}[k_x\cos(2\phi)+k_y\sin(2\phi)]^2+\\ &\frac{\hbar^2}{2m_2}[k_x\sin(2\phi)-k_y\cos(2\phi)]^2\\ =&[\frac{\hbar^2}{2m_1}\cos^2(2\phi)+\frac{\hbar^2}{2m_2}\sin^2(2\phi)]k_x^2+\\ &[\frac{\hbar^2}{2m_1}\sin^2(2\phi)+\frac{\hbar^2}{2m_2}\cos^2(2\phi)]k_y^2+\\ &(\frac{\hbar^2}{2m_1}-\frac{\hbar^2}{2m_2})\sin(4\phi)k_xk_y\ .\end{aligned} \tag{32}$$

For arbitrary direction $\mathbf{n}$=cos(θ)$\mathbf{x}$+sin(θ)$\mathbf{y}$ the electric field is applied along, at zero temperature, we have conductivity in matrix form contributed from valley K

$$\sigma^K = \frac{ne^2\tau}{m_1m_2}\begin{pmatrix} m_1\sin^2(\theta)+m_2\cos^2(\theta) & (m_1-m_2)\sin(2\theta)/2 \\ (m_1-m_2)\sin(2\theta)/2 & m_1\cos^2(\theta)+m_2\sin^2(\theta)\ , \end{pmatrix}$$

where $n = N/A$ is the carrier density, N is the number of bands (here $N = 1$) and A is the area of the unit cell. And from valley K'

$$\sigma^{K'} = \frac{ne^2\tau}{m_1m_2}\begin{pmatrix} m_1\sin^2(2\phi-\theta)+m_2\cos^2(2\phi-\theta) & -(m_1-m_2)\sin(4\phi-2\theta)/2 \\ -(m_1-m_2)\sin(4\phi-2\theta)/2 & m_1\cos^2(2\phi-\theta)+m_2\sin^2(2\phi-\theta) \end{pmatrix}\ ,$$

where the elements of the matrix are indexed by $\mathbf{n}$ and $\mathbf{n'}$ (the unit vector perpendicular to $\mathbf{n}$). The diagonal elements refer to longitudinal part and off-diagonal elements refer to the transverse part, or the Hall part of the conductivity. The total charge current $\sigma = \sigma^K + \sigma^{K'}$ then is

$$\sigma = \frac{ne^2\tau}{m_1m_2}\begin{pmatrix} m_1h_1(\psi,\theta)+m_2h_2(\psi,\theta) & -(m_1-m_2)\cos(2\phi)\sin(2\phi-2\theta) \\ -(m_1-m_2)\cos(2\phi)\sin(2\phi-2\theta) & m_1h_2(\psi,\theta)+m_2h_1(\psi,\theta) \end{pmatrix}$$

with $h_1(\psi,\theta) = [\sin^2(\theta)+\sin^2(2\phi-\theta)]$ and $h_2(\psi,\theta) = [\cos^2(\theta)+\cos^2(2\phi-\theta)]$. Since different valleys have different spin, we can define the spin current as $\sigma^S = \sigma^K - \sigma^{K'}$

$$\sigma^S = \frac{ne^2\tau}{m_1m_2}\begin{pmatrix} -(m_1-m_2)\sin(2\phi)\sin(2\phi-2\theta) & (m_1-m_2)\sin(2\phi)\cos(2\phi-2\theta) \\ (m_1-m_2)\sin(2\phi)\cos(2\phi-2\theta) & (m_1-m_2)\sin(2\phi)\sin(2\phi-2\theta) \end{pmatrix}\ .$$

The equivalent spin Hall angle is

$$S(\phi,\theta) = |\sigma^S_{\mathbf{nn'}}/\sigma_{\mathbf{nn}}| = \left|\frac{(m_1-m_2)\sin(2\phi)\cos(2\phi-2\theta)}{m_1[\sin^2(\theta)+\sin^2(2\phi-\theta)]+m_2[\cos^2(\theta)+\cos^2(2\phi-\theta)]}\right|\ . \tag{33}$$

When $\theta = \phi$

$$S(\phi) = \left|\frac{(m_1-m_2)\sin(2\phi)}{(m_1+m_2)-(m_1-m_2)\cos(2\phi)}\right|\ , \tag{34}$$

and the transverse spin current is pure, namely, there is no charge Hall current.

Now we Apply the above results to monolayer V_2Se_2O with $\phi = \pm\pi/4$, whose the band energy around the valley extrema centered at X and Y points have the form

$$\begin{aligned} E_X &= \frac{\hbar^2}{2m_1}k_x^2 + \frac{\hbar^2}{2m_2}k_y^2 \\ E_Y &= \frac{\hbar^2}{2m_1}k_y^2 + \frac{\hbar^2}{2m_2}k_x^2\ . \end{aligned} \tag{35}$$

For arbitrary direction $\mathbf{n}$=cos(θ)$\mathbf{x}$+sin(θ)$\mathbf{y}$ the electric field is applied along, at zero temperature, we have conductivity contributed from valley X

$$\sigma^X = \frac{ne^2\tau}{m_1m_2}\begin{pmatrix} m_1\sin^2(\theta)+m_2\cos^2(\theta) & (m_1-m_2)\sin(2\theta)/2 \\ (m_1-m_2)\sin(2\theta)/2 & m_1\cos^2(\theta)+m_2\sin^2(\theta) \end{pmatrix}$$

and from valley Y

$$\sigma^Y = \frac{ne^2\tau}{m_1m_2}\begin{pmatrix} m_1\cos^2(\theta)+m_2\sin^2(\theta) & -(m_1-m_2)\sin(2\theta)/2 \\ -(m_1-m_2)\sin(2\theta)/2 & m_1\sin^2(\theta)+m_2\cos^2(\theta) \end{pmatrix}\ .$$

The total charge current $\sigma = \sigma^K + \sigma^{K'}$ then is

$$\sigma = \frac{ne^2\tau}{m_1m_2}\begin{pmatrix} m_1+m_2 & 0 \\ 0 & m_1+m_2 \end{pmatrix}$$

Since different valleys have different spin, we can define

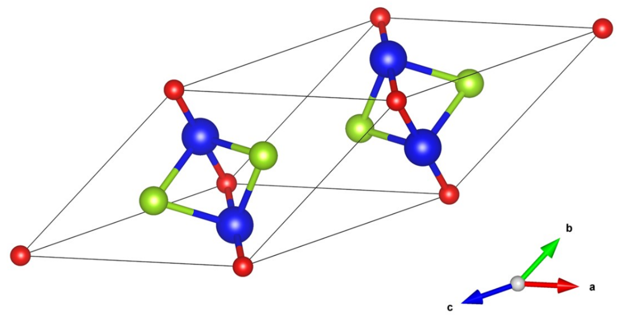

FIG. S1. Optimized primitive cell of bulk V_2Se_2O using optB88-vdW functional. The lattice constants are $a = b = c = 6.535$ Å with angle $\alpha = \beta = 145.4$ and $\gamma = 49.8$ deg.

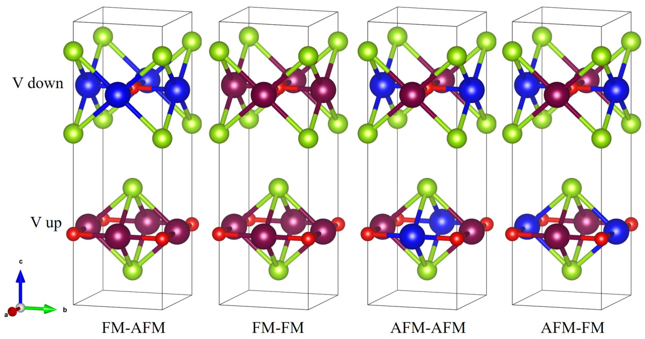

FIG. S2. Different magnetic order of bulk V_2Se_2O in a conventional cell, where FM-AFM refer to interlayer ferromagnetic and intralayer AFM coupled, similar as for other cases.

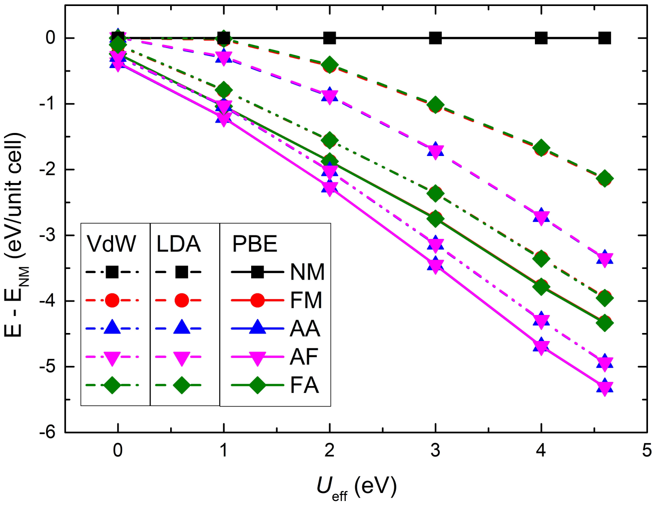

FIG. S3. Total energy difference ΔE, taking with respect to the NM order, for different functionals, different magnetic orders and at different U_{eff} of bulk V_2Se_2O. Ten atoms in one conventional cell. "A" or "F" is the short for AFM and FM. For the "$+U$" calculations, the simplified rotational invariant approach introduced by Dudarev et al. is used[7]. At $U_{eff} = 4.6$ eV, the band gap of monolayer V_2Se_2O is consistent with the one obtained by HSE06 calculations.

the spin current can as $\sigma^S = \sigma^K - \sigma^{K'}$

$$\sigma^S = \frac{ne^2\tau}{m_1 m_2}\begin{pmatrix} -(m_1-m_2)\cos(2\theta) & (m_1-m_2)\sin(2\theta) \\ (m_1-m_2)\sin(2\theta) & (m_1-m_2)\cos(2\theta) \end{pmatrix}$$

The equivalent spin Hall angle is

$$\begin{aligned} S(\theta) &= |\sigma^S_{\mathbf{nn}'}/\sigma_{\mathbf{nn}}| \\ &= \left|\sin(2\theta)\frac{m_1-m_2}{m_1+m_2}\right| . \end{aligned} \tag{36}$$

II. PROPERTIES OF BULK V_2SE_2O

A. Ground state determination

Powder samples of bulk V_2Se_2O had been synthesized recently, its lattice parameters were determined through x-ray diffraction (XRD) measurements[6]. The primitive cell is shown in Fig. S1, whose lattice constants are $a = b = c = 6.535$ Å and the angles between the lattice vectors are $\alpha = \beta = 145.4°$ and $\gamma = 49.8°$. For the calculations of the band structures and phonon spectrum of the bulk, the primitive cell was adopted.

However, in finding the ground state, the conventional cell was used in account of different magnetic orders, see Fig. S2. The conventional cell consists of two V_2Se_2O single layer with interlayer Se atoms AB stacked. Ferromagnetic (FM) state and three antiferromagnetic (AFM) states together with the nonmagnetic (NM) state are considered in the total-energy calculations, the results are listed in Fig. S3-4. With PBE functional at any value of the Hubbard U, it turns out that magnetic state is favored over the NM state for V_2Se_2O, especially when each single layer being AFM. The ground state is two AFM single layer are AFM coupled, i.e., the AFM-AFM state (3D Néel order) as shown in Fig. S2. To solid the conclusions, we have performed the same calculations using LDA functional and more properly, the VDW functional which including the corrections of the interlayer VDW interactions. We has also checked different implements of the Hubbard U, see Table S2, all the results are consistent.

B. Lattice constants

Lattice constants were then obtained by fully relaxing the primitive cell and transforming into the conventional cell in Table S1. Among the three functionals, PBE,optB88-vdW[9] and LDA, the VDW functional gives a best overall descriptions of the lattice constants; the PBE functional overestimates while the LDA functional underestimates the lattice constants. Hence later in the calculations of the phonon spectrum and band

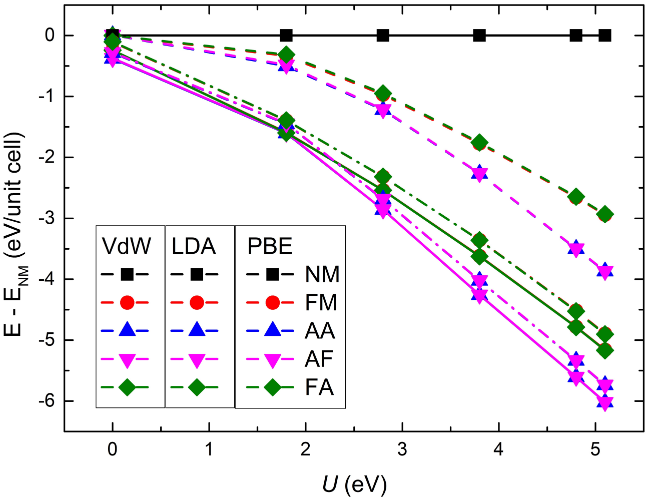

FIG. S4. Same as Fig. S3, but here the rotational invariant approach introduced by Liechtenstein et al[8]. is used for "$+U$" calculations. The Hund's rule coupling J is fixed at 0.8 eV. At $U = 5.1$ eV, the band gap of monolayer V_2Se_2O is consistent with the one obtained by HSE06 calculations.

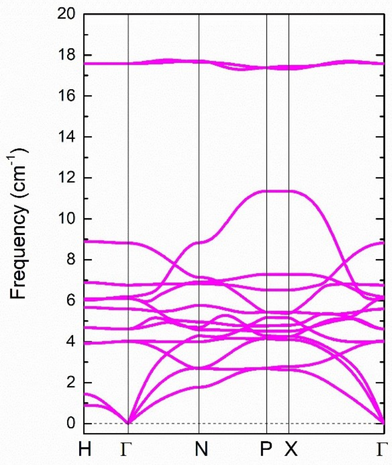

FIG. S5. Phonon spectrum of bulk V_2Se_2O at ground state (AFM-AFM state), calculated with optB88-vdW functional. A 3×3×3 supercell is used. Branches along the layered direction ΓH and PX disperse weakly, indicating weak interlayer couplings.

structures of the bulk, we shall adopt the optB88-vdW functional.

C. Phonon spectrum and band structures

Though bulk V_2Se_2O had been synthesized experimentally[6], we still calculate the phonon spectrum check the reliability of the calculations, see Fig. S5. No negative branch is observed, consistent with the experimental results. To be concrete, we have applied both DFPT and "finite difference (frozen phonon)" methods to obtain the phonon spectrum, which give the same distributions. Additionally, we find that in the phonon spectrum the dispersions along the interlayer direction, i.e., ΓH and PX branches, are weak, indicting weak interlayer couplings.

TABLE S1. Optimized lattice constants of bulk V_2Se_2O using different functionals. The optB88-vdW functional gives a best description in overall. Here AFM is the short for the ground 3D Néel state.

Functional	Magnetic order	a (Å)	c (Å)
	Not FM	3.887	11.700
PBE	NM	3.850	13.232
	FM	3.919	13.355
	AFM	3.897	13.144
vdW	NM	3.848	11.976
	FM	3.913	11.863
	**AFM**	**3.891**	**11.856**
LDA	NM	3.777	11.487
	FM	3.779	11.479
	AFM	3.786	11.434

Evolutions of the band structures with U values are shown in Fig. S6. With increasing the U, bulk V_2Se_2O experiences a phase transition from metal to insulator. Note in the experiments, V_2Se_2O is proposed to be a strongly correlated insulator with a Mott gap[6]. Therefore, finite U must be added in the calculations to reproduce the experimental results. The value of U is determined by fitting the band structures of monolayer V_2Se_2O to those obtained with more accurate hybrid functional calculations, see Fig. S6. The band structures are spin split along the interlayer direction ΓH and PX, and the diagonal ΓX (in the conventional cell). However, if two AFM V_2Se_2O single layer are FM coupled, the band structures are spin degenerate all through the BZ (not shown). Similar to the phonon spectrum, the dispersions of the energy bands along ΓH and PX are weak, indicting weak interlayer couplings. Hence we may expect that the bulk material may share similar properties as the monolayer.

D. Band structures of different magnetic orders

The band structures for different magnetic order of bulk V_2Se_2O is shown in Fig. S7. We can see that the paramagnetic, ferromagnetic orders are metallic while the AFM-AFM and AFM-FM (see Fig. S2) orders are insulating. This insulating behavior is consistent with the experimental observations[6], which indicates that V_2Se_2O prefers intra-layer AFM order.

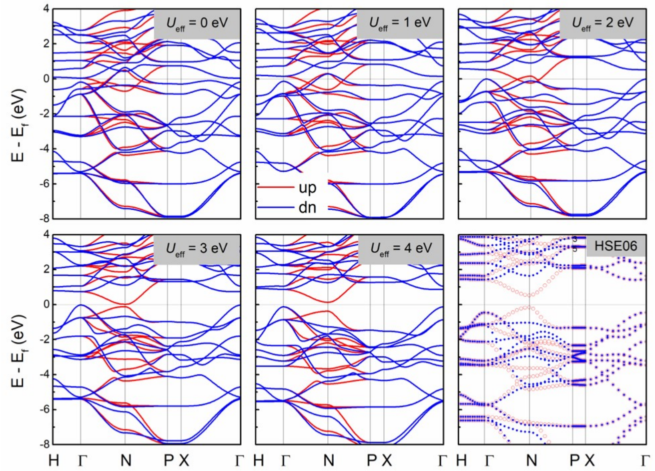

FIG. S6. Band structures of bulk V_2Se_2O at ground state (AFM-AFM state), calculated using optB88-vdW+U or HSE06 method. Similar to the phonon spectrum, branches along the layered direction ΓH and PX disperse weakly, indicating weak interlayer couplings.

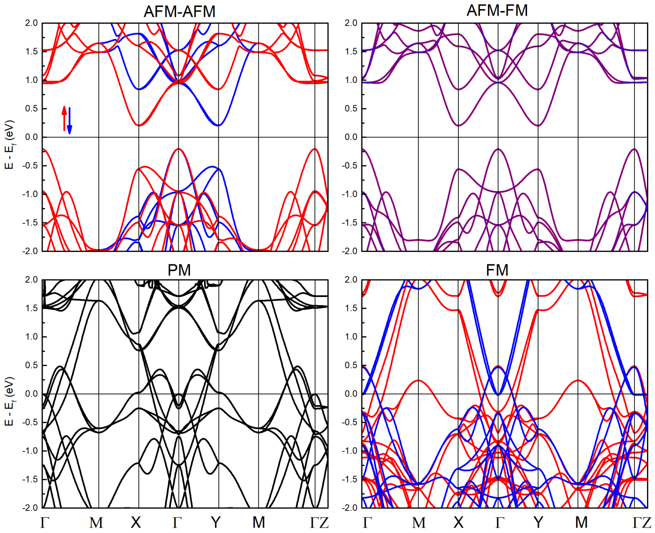

FIG. S7. Band structures of bulk V_2Se_2O with different magnetic orders. PM is for paramagnetic.

E. Band structures of V_2X_2O (X = O, S, Se or Te)

We calculate the band structures of a family of materials with common chemical formula V_2X_2O, where X= O, S, Se or Te, for both the bulk and monolayer, the results are shown in Fig. S8. As we can see, from O to Te, the band gap of V_2X_2O is getting smaller and smaller, so as the local magnetic moment. The band gap finally close for V_2Te_2O, which indicates that V_2X_2O is metallic, in consistent with the experimental observation[10].

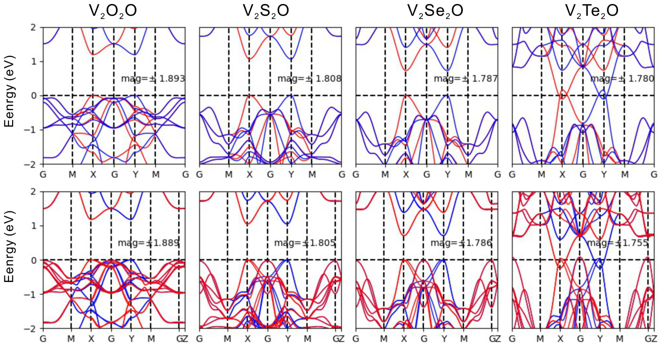

FIG. S8. Band structures of V_2X_2O (X = O, S, Se or Te). The first row is for the monolayer and the second row is for the bulk. Red (blue) is for spin up (down) polarization. The magnetic moment per V atom is indicated.

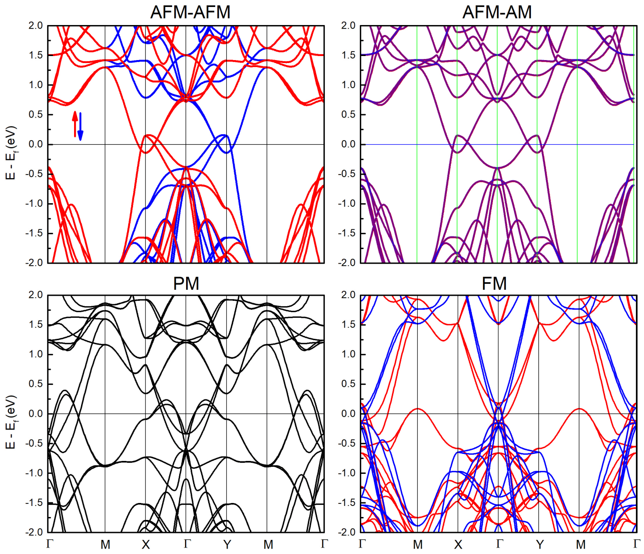

FIG. S9. Band structures of bulk V_2Te_2O with different magnetic orders. PM is for paramagnetic.

F. Band structures of bulk V_2Te_2O with different magnetic orders

The band structures for different magnetic order of bulk V_2Te_2O is shown in Fig. S9, as we can see, all the magnetic orders are metallic.

III. PROPERTIES OF MONOLAYER V_2SE_2O

A. Ground state and magnetic order

For the monolayer, we follow the same procedures of the bulk. First, we determine the ground state of the monolayer via total-energy calculations. Three magnetic states are considered including FM state, AFM Néel state and the stripy AFM state (see Fig. S14), the results are collected into Fig. S10. It turns out that the AFM

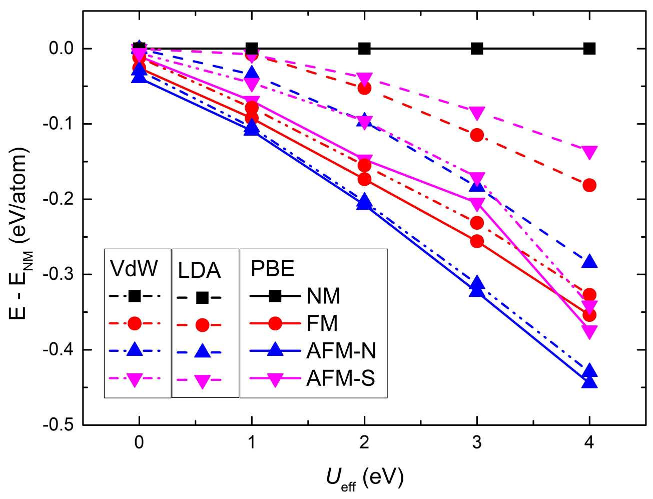

FIG. S10. Total energy difference ΔE, which is taken with respect to the NM order for different functionals, different magnetic orders and at different U_{eff} of monolayer V_2Se_2O. A 2×2 supercell with 20 atoms is used. AFM-N is the short for the Néel state, the ground state; and AFM-S is short for the stripy state, see Fig. S14. For the "$+U$" calculations, the simplified rotational invariant approach introduced by Dudarev et al. is used[7]. At U_{eff} = 4.6 eV, the band gap of monolayer V_2Se_2O is consistent with the one obtained by HSE06 calculations.

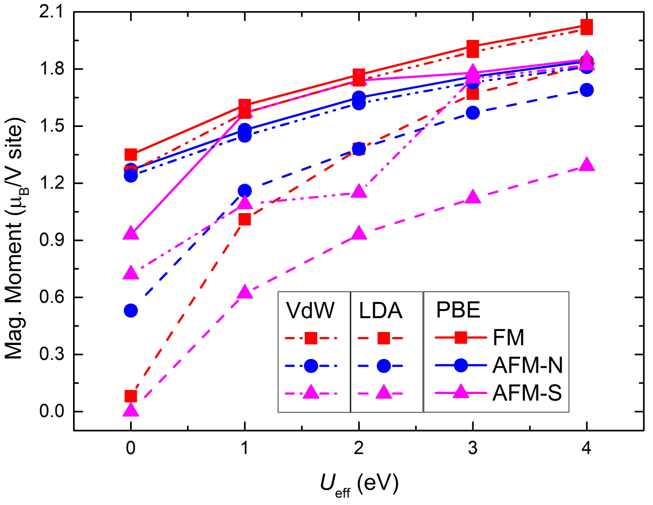

FIG. S11. Local magnetic moments of the V atoms, calculated with different functionals, different magnetic order and at different U_{eff} of monolayer V_2Se_2O.

Néel state has the lowest total energy and much more stable than other states at any value of U, especially when U is getting larger. The magnetization per V site is demonstrated in Fig. S11.

B. Lattice constants

We use two methods to obtain the optimized lattice constants of monolayer V_2Se_2O. First we use the equation of state to determine the them[11], the results are listed in Table S2; then we use VASP directly to get the lattice constants. The two methods give consistent results. For monolayer, PBE functional is used in further calculations since the VDW interactions does not enter into the monolayer. We had also performed the calculations with VDW functionals, the results are similar.

C. Magnetic properties

1. *Crystal-field-theory analysis*

From the chemical formula V_2Se_2O, V atom should roughly give 3 electrons to be in the V^{3+} state, and there will be two $3d$ electrons left that contribute to the local magnetic moments. Indeed, the magnetic moment obtained from first-principles calculations is 1.8 μB per V atom, indicating that the V^{3+} ion is in the spin-triplet configuration ($S = 1$), which is consistent with Hund's rule. In the rough octahedron-shape local crystal field of V atoms (Fig. S12), the $3d$ orbitals of V^{3+} will be first split into two-fold degenerate e_g and three-fold degenerate t_{2g} orbitals as shown in Fig. S12a. Different from the perfect octahedron crystal field, the differences of the O and Se atoms will further split the degeneracy within the two sets of degenerate orbitals. To be specific, the twofold degenerate e_g orbitals are split into two non-degenerate orbitals, and the t_{2g} orbitals are split into the twofold degenerate d_{xz} and d_{yz} states, and the non-degenerate d_{xy} state. Because the two $3d$ electrons are in the high spin state, one of them occupies the lowest d_{xy} orbital, and the other one occupies one of the doubly degenerate d_{xz} and d_{yz} orbitals, which will be further split into two non-degenerate orbitals due to Jahn-Teller effects[12]

TABLE S2. Optimized lattice constant and distance between the two Se layers of monolayer V_2Se_2O, using different functionals and for different magnetic order. Here AFM is the short for the ground 2D Néel state.The experimental value was taken from the bulk.

Functional	Magnetic order	a (Å)	d_{Se-Se} (Å)
	bulk	3.887	3.437
PBE	NM	3.869	3.472
	FM	3.929	3.411
	**AFM**	**3.908**	**3.443**
vdW	NM	3.845	3.493
	FM	3.903	3.426
	AFM	3.887	3.458
LDA	NM	3.789	3.420
	FM	3.789	3.420
	AFM	3.796	3.411

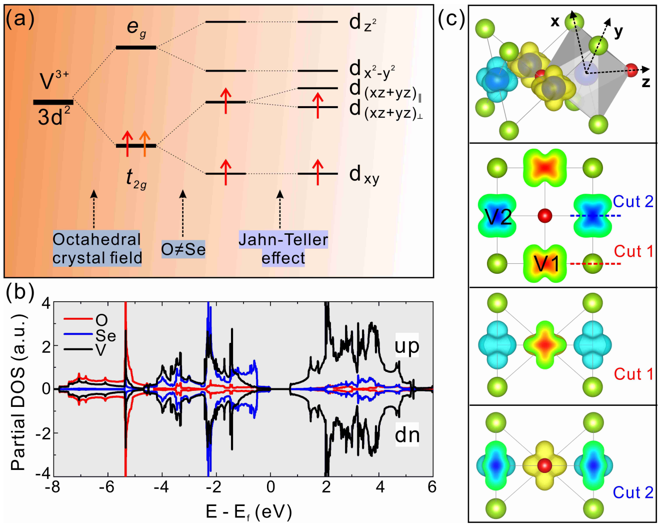

FIG. S12. Magnetic properties of monolayer V_2Se_2O. **a**, Schematic of $3d$ orbital splitting of V^{3+} under the local octahedral crystal field, where $(d_{xz} + d_{yz})_\parallel$ orbital is the in-plane (V-O plane) part of the linear combination of d_{xz} and d_{yz} orbitals and $(d_{xz} + d_{yz})_\perp$ the out-of-plane part, see Fig. S13 for details. **b**, Partial DOS of monolayer V_2Se_2O. **c**, Spin density distributions in real space, the V atoms are hidden in cut view.

The results from crystal field analysis are consistent with the first-principles calculations, and we indeed observed the splitting of $(d_{xz} + d_{yz})_\parallel$ or $(d_{xz} + d_{yz})_\perp$ orbitals (the subscript $\parallel$ and $\perp$ denotes the in-plane and out-of-plane part of the linear combination or d_{xz} and d_{yz} orbitals) in the site-projected DOS as shown in Fig. S13. Meanwhile, this can be further confirmed from the optimized lattice structure as shown in Fig. 2a, the four Se atoms surrounding a V atom form a rectangle instead of a square due to the Jahn-Teller distortion. The calculated ratio between the short side and the long side of the rectangle is about 0.88, which is consistent with the experimental value.

2. Site-projected DOS in local coordinates

From the lattice structure of monolayer V_2Se_2O we know that the V atoms are surrounded by a local octahedral, hence, to accurately describe the lm decomposed DOS, we adopt the local coordinates spanned by the octahedral to perform the site-projected calculations, see Fig. S13. Moreover, in doing this, we try to solid the understandings of the mirror symmetry of the monolayer. In details, for the V1 atom (see Fig. S12c), we have rotated the global **z** axis to the **b** direction and **y** to the **c** direction, the DOS are then shown in the top of each panel in Fig. S13; similarly, for the V2 atom, we have rotated the global **z** axis to the **a** direction and and **y** to the **c** direction, the DOS are then shown in the bottom of each panel in Fig. S13. In such coordinate systems, the d_{xz} and d_{yz} orbitals in the are transformed into $(d_{xz} + d_{yz})_\parallel$ the in-plane part and $(d_{xz} + d_{yz})_\perp$ the out-of-plane part with respect to the local octahedral coordinate. In a similar manner, the p_a or p_b orbital of Se are transformed from p_z orbital or the linear combinations or p_x and p_y orbitals. For an AFM compound, the structure can be separated into two sublattice, i.e., the spin-up sublattice which include the V1 atoms and spin-up components of the Se atoms (here we focus on the energies around the fermi level, contributions from the O atom are thus ignored), and the spin-down parts which include the V2 atoms and the spin-down components of the Se atoms. In such a partition we find that valleys in the valence bands are mainly from the p_z orbitals and valleys in the conduction bands originate from the $(d_{xz}+d_{yz})_\parallel$ orbitals. Based on these orbitals a four-band tight-binding model may be built to catch the physics of the valleys.

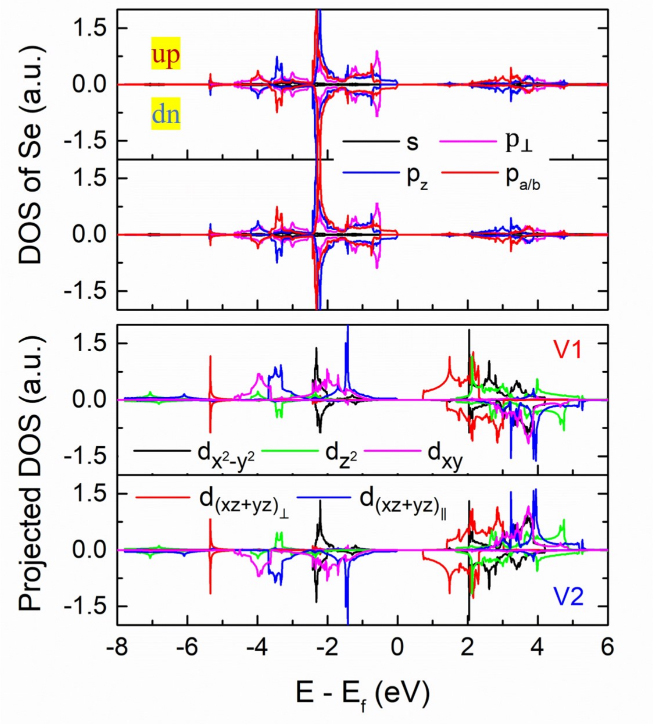

FIG. S13. DOS of monolayer V_2Se_2O. Top panel, lm decomposed DOS of Se, the upper and lower parts follow the same convention as the bottom panel. Bottom panel, Site projected DOS of V1 and V2 atoms as indicated in the inset image. To obtain the accurate lm decomposed and site projected DOS with respect to the local octahedral coordinate (we do not use this coordinate system since the quadrilateral formed by the four Se atoms do not arrange into a square), we adopt the local octahedral coordinate system during the calculations for DOS of V1 and V2 separately. For each calculation, the **z** axis (here the **xyz** axis are coincide with the **abc** axis of the lattice) is rotate to coincide with the principal axis of the corresponding octahedron (see the text), and the **y** axis along the out-of-plane direction. All the subscript xyz in the image are for the local octahedral coordinate.

3. Superexchange interactions

With the above orbital splitting in mind, we begin to study the exchange interactions between the V atoms to understand the antiferromagnetism in monolayer V_2Se_2O. First, the p electrons of O atoms are not expected to be irrelevant since their energy are far away from the Fermi level (see partial DOS in Fig. S12b). Therefore, we focus on the exchange interactions including the nearest neighbors (NN) and next-nearest neighbors (NNN) within the V and Se atoms. For the NN exchange interactions, the main contribution comes from the superexchange via the Se atoms. We may treat this superexchange as an effective exchange between the V1 and V2 atoms (see Fig. 12c) after considering the hybridization of the p orbitals of Se and $3d$ orbitals of V. As illustrated in the top view of the spin density distribution in Fig. S12c, the occupied $(d_{xz}+d_{yz})_{\|}$ orbitals of V1 and V2, are directed head-to-head, which results into large overlap of the two orbitals. Therefore, the effective NN exchange between V1 and V2 favors AFM and should be strong according to the general Goodenough-Kanamori-Anderson (GKA) rules[13–15]. Contributions from other orbitals can be neglected since their overlaps are comparably small. For the NNN exchange interactions, similar analysis shows that the superexchange via Se atoms also prefers AFM, but they have limited influence on the overall exchange interactions because the distance for the effective exchange is much longer. In total, monolayer V_2Se_2O prefers AFM and the exchange interactions is dominated by the NN superexchange.

The above analysis is consistent with the first-principles calculations. Total energy calculations show that the ground state of monolayer V_2Se_2O is AFM of the 2D Néel type, which is much more stable than paramagnetic and other magnetic states and independent on the exchange-correlation functionals used in calculations (see more details in Fig. S10). To solid the results, we quantitatively extract the exchange coupling parameters based on a Heisenberg model using both supercell (Fig. S14) and the "frozen magnon" methods[12, 16] (Table S3).

4. Parameters of the Heisenberg model

We adopt the following Heisenberg model:

$$H=-\sum_{\langle ij\rangle} J_1\mathbf{S_i}\mathbf{S_j}-\sum_{\langle kl\rangle} J_2^{O}\mathbf{S_k}\mathbf{S_l}-\sum_{\langle kl\rangle} J_2^{Se}\mathbf{S_k}\mathbf{S_l}\ , \quad (37)$$

where $\langle ij\rangle$ the nearest neighbors and $\langle kl\rangle$ the next-nearest neighbors, $\mathbf{S}$ is the net spin of each V atom. Assuming that the V atoms are polarized parallel along the $\mathbf{c}$ direction, then the $\mathbf{S}$ can be simply replaced with its magnitude S (Ising models). We use two methods to obtain these Heisenberg parameters. The first method is based on the assumption that energy difference between

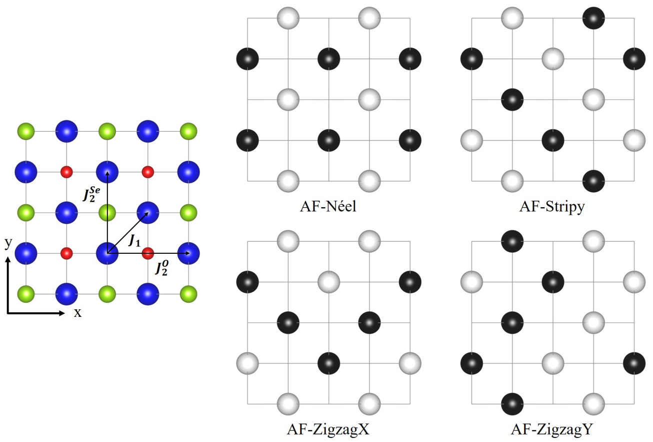

FIG. S14. Spin configurations which are insulating for the calculations of Heisenberg exchange parameters, 2×2 supercell which contains 8 V atoms. Here we denote the exchange interactions which path through O atoms as J_2^O, path through Se atoms as J_2^{Se}.

different magnetic oder only comes from the Heisenberg exchange energy, that is the total energy of any magnetic state can be written as two parts:

$$E_{Morder}=E_0+E_{Heisenberg}\ , \quad (38)$$

where E_0 is the background energy which is a constant for all magnetic order;the second term is nothing but the Heisenberg exchange energy. Note that the assumption is only valid when all the magnetic order are in the same phase (metallic or insulating); or no more precisely, no other interactions except for the exchange interactions that vary with magnetic order. Since there are three unknown Heisenberg parameters, four magnetic order need to be considered, see Fig. S14. Taking the ground state (2D Néel) as reference, the total energy difference between the AF-stripy state is

$$E_{SN}=16(-J_1+J_2^O+J_2^{Se})S^2, \quad (39)$$

between the AF-ZigzagX state is

$$E_{XN}=8(-2J_1+2J_2^O+J_2^{Se})S^2, \quad (40)$$

between the AF-ZigzagY state is

$$E_{YN}=8(-2J_1+J_2^O+2J_2^{Se})S^2. \quad (41)$$

After acquiring the total energy difference from DFT calculations, the Heisenberg parameters can then be determined through above equations. We have performed the calculations using both a 2×2 and 4×4 supercell, the results are listed in Table S3.

The second method to obtain the Heisenberg parameters is the spin-spiral calculations with "frozen magnon" method, details can be find in ref[12, 16]. The method has some advantages over the last one since there are only magnon excitations, one needs not to consider the phases

of the states as before. The total energy calculations are performed using all-electron full-potential linearised augmented-planewave (FP-LAPW) ELK code[17]. The cone angle is set as $\cot(\theta) = 3$ rad. A 4×4 q-mesh over the 2D BZ is adopt for back-Fourier transformation and a 8×8 k-mesh is taken for total energy calculations. The total energy (epsengy tag) and RMS change (epspot tag) convergence criteria is set at 10^{-6} and 2×10^{-6} Hartree respectively. SOC has not been included.

Inconsistence is encountered for the determination of J_2, which indicates that the Heisenberg model maybe too simple to model our system. More terms such as the $p-d$ mixing between the p orbitals of Se and d orbitals of V, which may play an important role in lowering the total energy, should be included. While all the calculations show that J_1 is negative and its value is much larger than J_2, strongly suggest that the AFM order in the compound is robust.

In addition, we have also calculated that there is a weak easy-plane anisotropy on the order of ∼0.05 meV per V atom for monolayer V_2Se_2O. Such a small anisotropy may not be able to stabilize a long-range order at high temperature according to Mermin–Wagner theorem[18]. However, as shown in Fig. 2a, V_2Se_2O monolayer is not strictly 2D, but consists of two Se layer and one V-O layer, the constraint imposed by the theorem may not be so strong in our structure. Besides, some other long-range order associated with continuous symmetry breaking such as superconductivity[7] has been observed in 2D materials as well. Therefore, we believe that at least within some length scale, the stable 2D Néel order of V_2Se_2O predicted in the present paper can be maintained experimentally.

5. *Discussion*

Here we give a further discussion on the antiferromagnetism in monolayer V_2Se_2O. Based on our systematical calculations and theory analysis, we believe that it is highly possible that monolayer V_2Se_2O could be a 2D AFM material to realize the C-paired SVL. The reasons are as following:

1. The authors of ref[6] observed a log(1/T) temperature dependence of the resistance for V_2Se_2O, which demonstrates that V_2Se_2O is insulating. In addition, they had also extracted a value of 0.569 μ_B magnetic moment for each V atom in V_2Se_2O from the magnetic susceptibility data, which confirms the existence of local magnetic moments. On the contrary, V_2Te_2O was found to be metallic and has no observable local magnetic moments in ref[10].

2. We did systematic calculations for the electronic and magnetic structures of bulk V_2X_2O (Fig. S8), where X=O, S, Se or Te. We found the difference between V_2Se_2O and V_2Te_2O can be attributed to the different crystal field effect, which is stronger in V_2Se_2O due to the larger electronic negativity of Se and shorter V-Se bonds. The large crystal field effect in V_2Se_2O also leads to stronger exchange interactions between the local magnetic moments (∼85 meV), which forces the local magnetic moments to form the in-plane AFM alignment.

3. Moreover, for V_2Se_2O, the band structures strongly depend on the magnetic order. As shown in Fig. S7, V_2Se_2O is metallic with large Fermi surface in both FM and PM cases, and it is insulating only when the local magnetic moments of monolayer V_2Se_2O has an in-plane AFM alignment. However, as shown in Fig. S9, V_2Te_2O is always metallic even the local magnetic moments have an in-plane AFM alignment.

4. Regardless of the strong in-plane AFM ordering, bulk V_2Se_2O only exhibit paramagnetic behavior, which is due to the small interlayer coupling (∼0.11 meV) and in-plane magnetic anisotropy (∼0.05 meV).

5. Note that paramagnetic bulk does not guarantee paramagnetic monolayer. For example, bulk VSe_2 is paramagnetic while monolayer VSe_2 is demonstrated to be room-temperature ferromagnetic[19].

Therefore, we can conclude that the most possible scenario for V_2Se_2O is that it is paramagnetic for the bulk, but AFM for the monolayer. In addition, even if monolayer V_2Se_2O is PM, one can use an external magnetic field to stabilize the AFM order which is feasible since the AFM interaction here is very strong (∼85 meV)

TABLE S3. Heisenberg exchange parameters of monolayer V_2Se_2O. Unit for energy difference and exchange parameters is meV. Note for 4×4 supercell, the AF-Stripy state does not converge, an alternative state which is similar to AF-Zigzag state with the size of the zigzag increase by one V atom is calculated. Spin-spiral calculations are performed using "frozen magnon" method.

Model & Method	J_1	J_2^O	J_2^{Se}
2×2 supercell	-38.5	10.0	18.0
4×4 supercell	-66.5	-8.7	-0.7
Spin-spiral	-149.9	-4.7	-36.9

D. Band structures of monolayer V_2Se_2O obtained with different functionals

We first apply "DFT+U" method to calculate the band structures[8], evolutions of the band structures with respect to U values while fixing Hund's rule couplings $J = 0.8$ eV are shown in Fig. S15. Note we have also repeated the calculations with the simplified "DFT+U" method proposed by Dudarev et al.[7], the results are

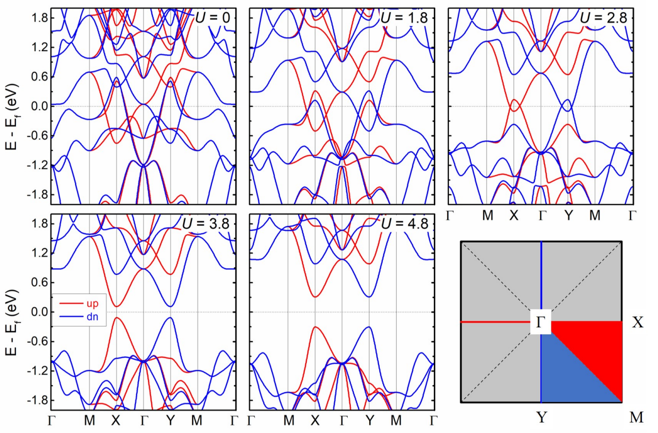

FIG. S15. Band structures of monolayer V_2Se_2O at different U, the Hund's rule coupling J is fixed as 0.8 eV. Fermi level is set at the middle of the band gap if there is one. Bottom right panel, schematic of the 2D square Brillouin zone. The red and blue triangles denote the integrated areas for the DOS in the following figure.

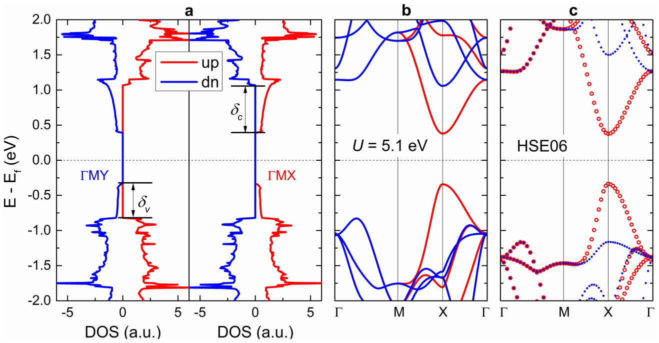

FIG. S16. **a**, Total DOS of monolayer V_2Se_2O. Left (right), integrated over the triangle area ΓMY (ΓMX) as indicated in Figure S11. Hidden information about the spin splitting in the total DOS can be uncovered in this way. δ_v (δ_c) indicates the exchange splitting gap in valence (conduction) bands. **b**, Band structures at U = 5.1 eV and J = 0.8 eV. By fitting band gap with the one obtained from HSE06 calculations, the Hubbard U can be determined. **c**, Band structures calculated with more accurate HSE06 functional. The exchange splitting is more prominent.

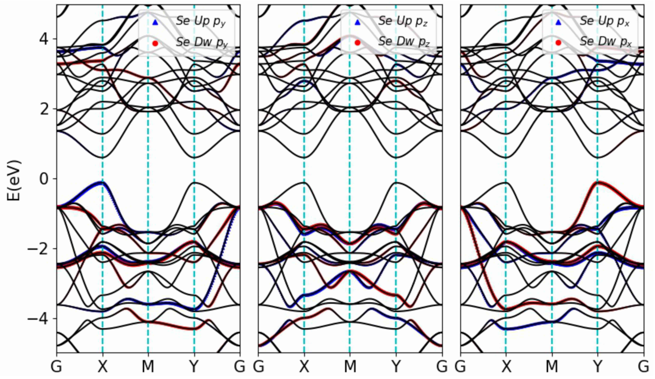

FIG. S17. Projected band structures for the Se atoms of monolayer V_2Se_2O

similar. With increasing the U, monolayer V_2Se_2O experience a phase transition from metal to insulator,which is a typical behavior of Mott insulator. The SVL also becomes clear as U getting larger. To show that SVL does exist in monolayer V_2Se_2O, we integrated out the DOS over the area of the first BZ which covers only single valley, i.e., ΓMY and ΓMX respectively as shown in Fig. S16a. We can see that valleys in the conduction bands and valence bands are both spin-polarized, which indicates the SVL.

We have also calculated the band structures with more accurate HSE06 functional[20], see Fig. S16c, which has similar dispersions with those obtained with "DFT+U " method at large U. Specifically, when U = 5.1 eV, the two sets of band structures are highly similar. Therefore, in the following calculations ,we adopt the more calculationally cheaper "DFT+U " method with U = 5.1 eV and J = 0.8 eV.

From Fig. S16a-b we can see that the spin-split gaps δ_v = 0.83 eV and δ_c = 0.88 eV (see Fig. S16a), which are even larger than the band gap and much larger than the SOC-induced spin splitting in TMDs. This indicates that for monolayer V_2Se_2O, both electrons and electrons can serve as charge carriers with long spin life time.

E. Projected band structures

In Fig. S17-18 the projected band structures of monolayer V_2Se_2O are shown, spin polarization is included. Since the orbitals of O atoms lie deep in energy, hence do not show here. For the Se and V atoms, as we can see that the valence valleys are mainly contributed from the p orbitals of Se atoms (and together part of the d_{xy} of V atoms), where the spin-up branch of p_y orbitals constitute of the X valley and the spin-down branch of p_x orbitals constitute the Y valley. The conduction valleys are contributed from V atoms. Specifically, the X valley is made from the spin-up branch of d_{yz} orbital of V1 and Y valley is made from the spin-down branch of d_{xz} orbital of V2. We also find that SOC have little effects on the valley extrema as one can see in Fig. S19. The spin polarizations around the valley extrema keep intact even including SOC as we have checked the spin textures.

F. Strain effects on the band structures and effective mass

We have also studied the deformations of the band structures under uniaxial strain, see Fig. S20, the band gap getting larger under tensile strain whereas becoming smaller when compressive strain is applied. The influence of the strain to the relative shift in energy of the conduc-

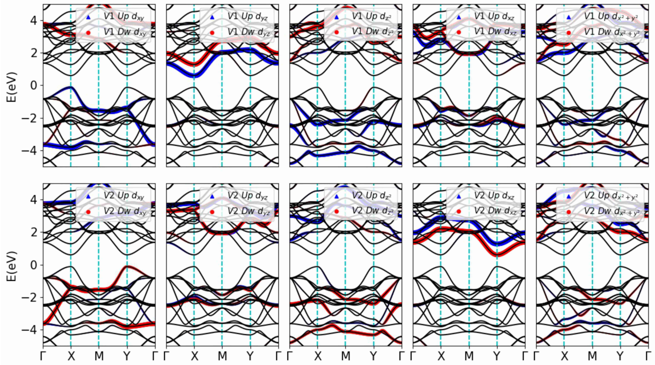

FIG. S18. Projected band structures for the V atoms of monolayer V_2Se_2O

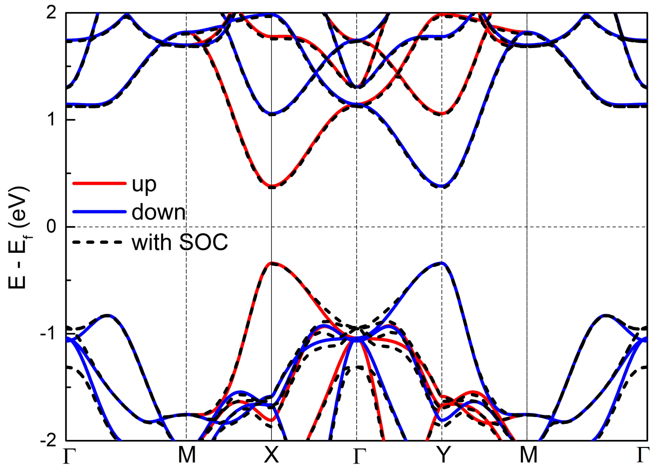

FIG. S19. Band structures of monolayer V_2Se_2O with (dash line) and without SOC (solid lines). The SOC have little effects on the valleys

tion bands is not obvious, while for the valence bands, the X valley is pushed lower in energy at compressive strain while getting higher at tensile. This imbalance of the energy shift of the two valleys is the origin of the piezomagnetic effect after doping the sample.

After obtaining the strained band structures, the effective masses of the valence valley extrema under different strain can calculated directly with $m_{\mu\nu} = \hbar^2/\frac{\partial E^2}{\partial k_\mu \partial k_\nu}$. We calculated m_{xx} and m_{yy} of both the X and Y valleys of monolayer V_2Se_2O, which are shown in Fig. S21. For uniaxial strain along **a** (or **x**) direction, k_y keeps unchanged, hence the effective mass m_{yy} of the both the X and Y valleys should show weak dependence on the strain; while k_x is proportional to the strength of the uniaxial strain. Therefore, the effective mass m_{xx} of both the X and Y valleys should be almost linearly dependent on the external strain. These are what we indeed see in Fig. S21. Since the DOS of a 2D parabolic band is linearly dependent on the effective mass, the change of the effective mass would leads to increasing or decreasing of the DOS, and therefore affects the behaviors of the strain-induced magnetizations.

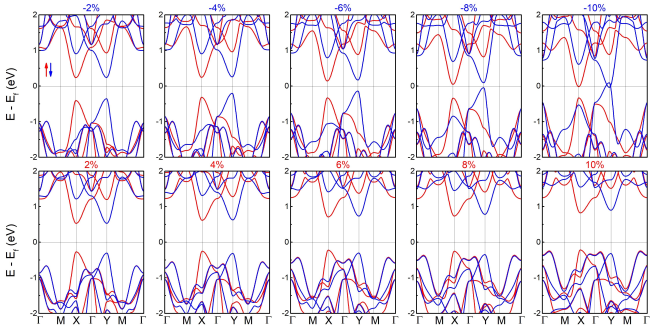

FIG. S20. Band structures at different uniaxial strain of monolayer V_2Se_2O. Taking at $U = 5.1$ eV and $J = 0.8$ eV, strain applied along **a** direction.

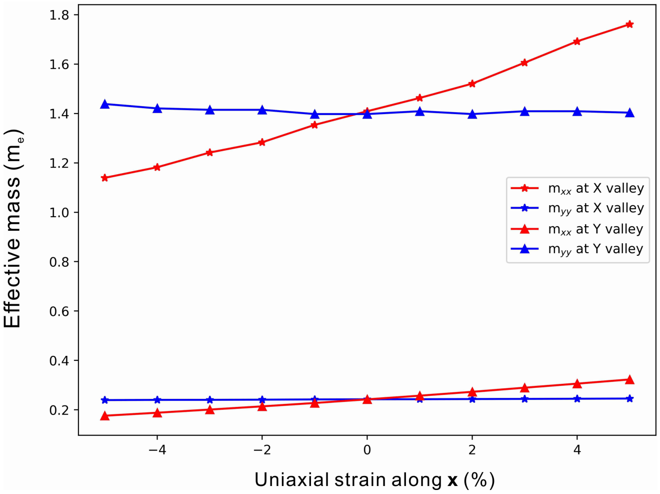

FIG. S21. Effective masses of the valence valleys extrema as a function of uniaxial strain along **x** (or **a**) direction. m_e is the mass of the electron.

G. Magnetization induced by uniaxial strain along b direction

The calculated magnetizations induced by uniaxial strain along **a** axis are present in the main text. Here similar results can be found for the strain along **b** (or **b**) in Fig. S22, the only difference is the induced magnetizations change sign, which is as expected due to the diagonal mirror symmetry. The induced magnetizations were first obtained with a very dense k-mesh, directly using VASP or interpolated with *wannier*90 code[21]. The results were further verified with a mixed k-grid method, namely, around the valleys a denser k-mesh is used. All the results are consistent.

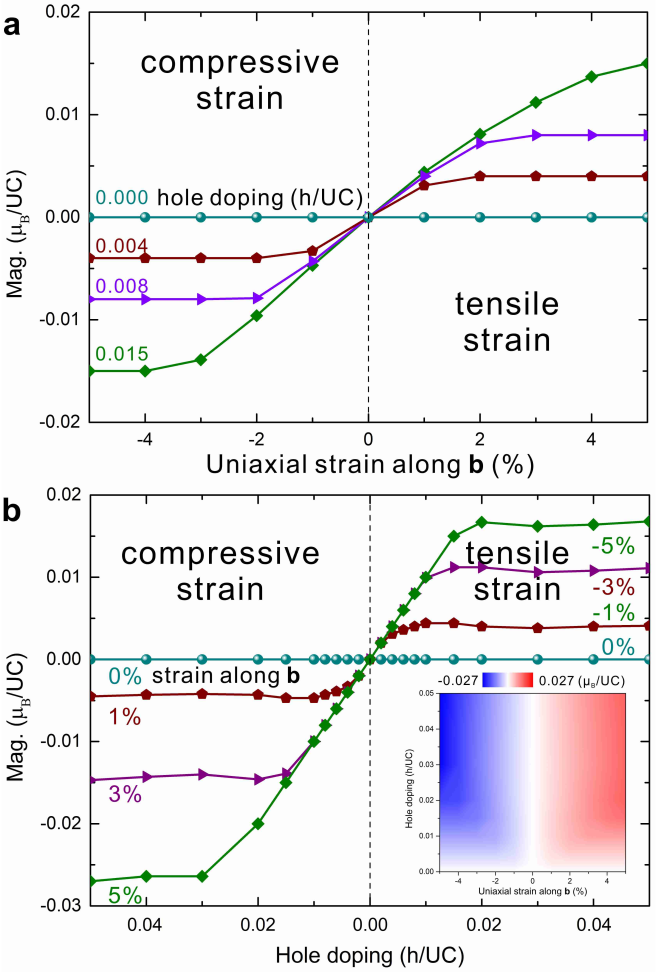

FIG. S22. Magnetization induced by uniaxial strain along **b** direction for hope-doped monolayer V_2Se_2O. **a**, For a given hole density, the net magnetization increases linearly with strain but has opposite direction for compressive and tensile strains in the region of small strains. When strain is large enough, all the carriers are polarized, and the net magnetization saturates. **b**, For any given strain, the magnetization is always equal to the number of holes per unit cell when the doping is light. However, for heavy doping, the magnetization is almost a constant which depends on the strain. Inset is the diagram of strain-induced net magnetization for different hole densities.

H. Calculations of the DC spin current of monolayer V_2Se_2O

After obtaining the tight-banding Hamiltonian with the interface of *wannier*90 code[21] to VASP, the conductivity contributed from each valley are calculated directly with Eq. (26). The electronic temperature and relaxation time are set as $T = 150$ K and $\tau = 10^{-13}$ s respectively. A 300×300 k-mesh is used for the integration over the BZ. Different parameters were tested which gave similar results.

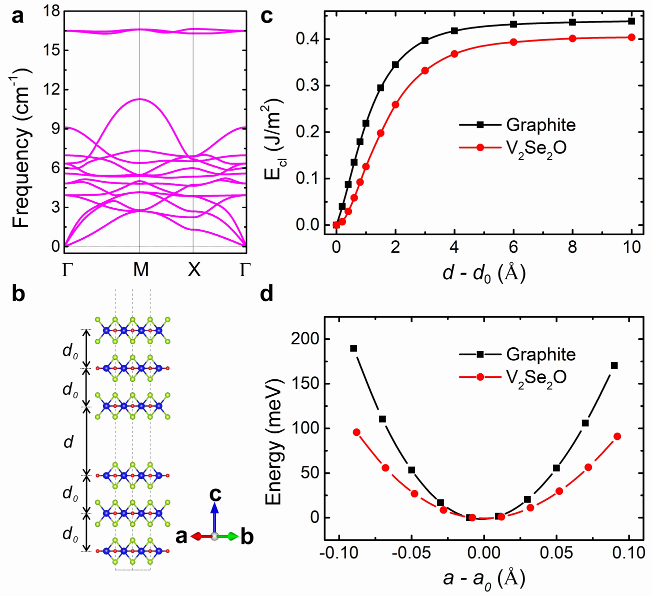

FIG. S23. Mechanical and dynamic properties of monolayer V_2Se_2O. **a**, Phonon spectrum of monolayer V_2Se_2O. **b**, Lattice model for the exfoliating procedure, where d_0 is the equilibrium interlayer distance and d, the separation of the two fractured parts. **c**, Cleavage energy of graphite and V_2Se_2O as a function of the separation $d - d_0$. **d**, Variation of total energy with the 2D lattice constants for a single layer graphite and V_2Se_2O.

I. Dynamical stability and mechanical properties

Now we turn to the experimental preparation of monolayer V_2Se_2O, which could be checked by the dynamical stability, cleavage energy and in-plane stiffness. As shown in Fig. S23a, there is no negative branch signified in the phonon spectrum, indicating the structure of monolayer V_2Se_2O is dynamically stable. In addition, for a 2D material to be easy to exfoliate for further experimental investigation, there are two requirements[22]: i) small cleavage energy, which gives a quantitative description of weather it is easy to exfoliate nanosheets from bulk material. ii) strong in-plane stiffness of the corresponding 2D lattice, which enable us to obtain large-scale flakes which is needed for experimental detection.

The cleavage energy is calculated by determining the total energy difference (per unit area) of the fractured structure as a function of the separation d, with respect to the equilibrium position d_0[23]. The results are illustrated Fig. S23b-c. The cleavage energy of V_2Se_2O increases rapidly with increasing d at first, then gradually converges to 0.40 J/m^2, which is slightly smaller than that of graphite (0.44 J/m^2[24]) calculated within the same scheme. We have also calculated the cleavage energy of the monolayer (bilayer) sheets and all the results are consistent (0.38 (0.40), 0.37 (0.41) J/m^2 for V_2Se_2O and graphite). Similar calculations have given 0.24, 0.35 and 0.38 J/m^2 for the widely studied 2D vdW materials

transition metal tri-chalcogenides class $CrCTe_3$[25],[12] $CrSiTe_3$ and $CrGeTe_3$[22] respectively and 0.27 J/m^2 for MoS_2[26]. All the calculations indicate that monolayer V_2Se_2O should be easily prepared through the exfoliation as other well studied 2D materials.

The in-plane stiffness is given by 2D Young's modulus which can be evaluated according to the equation

$$Y_{2D} = A_0\left(\frac{\partial^2 E}{\partial A^2}\right)_{A_0} = \frac{1}{4}\left(\frac{\partial^2 E}{\partial a^2}\right)_{a_0}, \tag{42}$$

where E is the total energy, a is the 2D lattice parameter and A is the corresponded surface area. From the change of total energy with respect to 2D lattice constant (Fig. S23d), we obtained Y_{2D}= 94.5 N/m for single layer V_2Se_2O and Y_{2D}= 212.7 N/m for graphene. The in-plane stiffness of V_2Se_2O is about 60% of graphene, the well-known ultra-strong material. This rather high in-plane stiffness means that large-scale V_2Se_2O monolayers with the free-standing structure could be expected to obtain during the exfoliation.

[1] G. L. Bir and G. E. Pikus, "Symmetry and strain-induced effects in semiconductors," (Wiley New York, 1974) p. 301.

[2] T. B. Bahder, Physical Review B **41**, 11992 (1990).

[3] Y. Zhang, Physical Review B **49**, 14352 (1994).

[4] N. W. Ashcroft and N. D. Mermin, "Solid state physics," (Beijing World Publishing Corporation, 1976) pp. 250, Eq. 13.25.

[5] N. W. Ashcroft and N. D. Mermin, "Solid state physics," (Beijing World Publishing Corporation, 1976) pp. 251, footnote 17.

[6] H. Lin, J. Si, X. Zhu, K. Cai, H. Li, L. Kong, X. Yu, and H.-H. Wen, Physical Review B **98**, 075132 (2018).

[7] S. Dudarev, G. Botton, S. Savrasov, C. Humphreys, and A. Sutton, Physical Review B **57**, 1505 (1998).

[8] A. Liechtenstein, V. Anisimov, and J. Zaanen, Physical Review B **52**, R5467 (1995).

[9] J. Klimeš, D. R. Bowler, and A. Michaelides, Physical Review B **83**, 195131 (2011).

[10] A. Ablimit, Y.-L. Sun, E.-J. Cheng, Y.-B. Liu, S.-Q. Wu, H. Jiang, Z. Ren, S. Li, and G.-H. Cao, Inorganic chemistry **57**, 14617 (2018).

[11] F. Birch, Physical review **71**, 809 (1947).

[12] K. I. Kugel and D. Khomskiĭ, Soviet Physics Uspekhi **25**, 231 (1982).

[13] J. B. Goodenough, Journal of Physics and Chemistry of Solids **6**, 287 (1958).

[14] J. Kanamori, Journal of Physics and Chemistry of Solids **10**, 87 (1959).

[15] P. W. Anderson and H. Hasegawa, Physical Review **100**, 675 (1955).

[16] M. Ležaić, P. Mavropoulos, G. Bihlmayer, and S. Blügel, Physical Review B **88**, 134403 (2013).

[17] K. Dewhurst, S. Sharma, and et. al., "The elk code," `http://elk.sourceforge.net/`.

[18] N. D. Mermin and H. Wagner, Physical Review Letters **17**, 1133 (1966).

[19] M. Bonilla, S. Kolekar, Y. Ma, H. C. Diaz, V. Kalappattil, R. Das, T. Eggers, H. R. Gutierrez, M.-H. Phan, and M. Batzill, Nature nanotechnology **13**, 289 (2018).

[20] J. Heyd, G. E. Scuseria, and M. Ernzerhof, The Journal of chemical physics **118**, 8207 (2003).

[21] A. A. Mostofi, J. R. Yates, Y.-S. Lee, I. Souza, D. Vanderbilt, and N. Marzari, Computer Physics Communications **178**, 685 (2008).

[22] X. Li and J. Yang, Journal of Materials Chemistry C **2**, 7071 (2014).

[23] J. He, S. Ma, P. Lyu, and P. Nachtigall, Journal of Materials Chemistry C **4**, 2518 (2016).

[24] M. A. McGuire, H. Dixit, V. R. Cooper, and B. C. Sales, Chemistry of Materials **27**, 612 (2015).

[25] S. Chabungbam and P. Sen, Physical Review B **96**, 045404 (2017).

[26] T. Björkman, A. Gulans, A. V. Krasheninnikov, and R. M. Nieminen, Physical review letters **108**, 235502 (2012).
