# Supplementary material for: Multifunctional antiferromagnetic materials with giant piezomagnetism and noncollinear spin current
Source: Nat Commun. 2021 May 14;12:2846. doi: 10.1038/s41467-021-23127-7 (PMC8121910; doi:10.1038/s41467-021-23127-7)
Supplement: Supplementary file 2 — Peer Review File [file 41467_2021_23127_MOESM2_ESM.pdf]

Editorial Note: This manuscript has been previously reviewed at another journal that is not operating a transparent peer review scheme. This document only contains reviewer comments and rebuttal letters for versions considered at *Nature Communications*. Mentions of prior referee reports have been redacted.

Reviewer #2:
Remarks to the Author:
The article is well written and I sincerely believe that a timely publication of it will be beneficial to the scientific community. I recommend the publication of it in its current form in Nature Communications.

Reviewer #3:
Remarks to the Author:
I am happy with the changes that authors have made and think the manuscript is much improved; they have proposed a new and novel kind of spin-valley locking mechanism (C-paired SVL).

I can, therefore, recommend the publication of this manuscript in Nature Communications.
